# White Matter Integrity Involvement in the Preclinical Stage of Familial Creutzfeldt–Jakob Disease: A Diffusion Tensor Imaging Study

**DOI:** 10.3389/fnagi.2021.655667

**Published:** 2021-05-19

**Authors:** Donglai Jing, Yaojing Chen, Kexin Xie, Yue Cui, Chunlei Cui, Li Liu, Hui Lu, Jing Ye, Ran Gao, Lin Wang, Zhigang Liang, Zhanjun Zhang, Liyong Wu

**Affiliations:** ^1^Department of Neurology, Xuanwu Hospital, Capital Medical University, Beijing, China; ^2^Department of Neurology, Rongcheng People’s Hospital, Hebei, China; ^3^State Key Laboratory of Cognitive Neuroscience and Learning, Beijing Normal University, Beijing, China; ^4^Department of Nuclear Medicine, Xuanwu Hospital, Capital Medical University, Beijing, China

**Keywords:** Creutzfeldt–Jakob disease, preclinical stage, diffusion tensor imaging, tract-based spatial statistics, white matter

## Abstract

**Objective:**

The objective of the study was to explore patterns of white matter (WM) alteration in preclinical stage familial Creutzfeldt–Jakob disease (fCJD) using diffusion tensor imaging (DTI).

**Methods:**

Seven asymptomatic carriers of the *PRNP* G114V mutation and six non-carriers were recruited from the same fCJD kindred and follow-up obtained from all asymptomatic carriers and two non-carriers 2 years later. Overlapping WM patterns were also explored in asymptomatic carriers and symptomatic CJD patients. All participants underwent clinical and neuropsychological assessments and DTI at baseline and follow-up. DTI data were subjected to whole-brain voxel-wise analysis of fractional anisotropy (FA) and mean diffusivity (MD) in WM using tract-based spatial statistics. Three comparisons were conducted: baseline carriers against non-carriers (baseline analysis), changes after 2 years in carriers (follow-up analysis), and differences between patients with symptomatic CJD and healthy controls (CJD patient analysis).

**Results:**

Neither carriers nor non-carriers developed any neurological symptoms during 2 years of follow-up. Baseline analysis showed no differences between the carrier and non-carrier groups in MD and FA. Follow-up analysis showed significantly increased MD in multiple WM tracts, among which increased MD in the bilateral superior longitudinal fasciculus, bilateral anterior thalamic radiation, bilateral cingulate gyrus, and left uncinate fasciculus overlapped the patterns observed in patients with symptomatic CJD.

**Conclusion:**

Changes in integrity within multiple WM tracts can be detected during the preclinical stage of fCJD.

## Introduction

Creutzfeldt–Jakob disease (CJD) is a rare, fatal neurodegenerative disorder characterized by rapidly progressive dementia, motion disturbances, and akinetic mutism. Familial CJD (fCJD), which is caused by mutations in the prion protein gene, *PRNP*, and sporadic CJD (sCJD) are two major forms of CJD that share similar pathophysiological features (Jeong and Kim, 2014). Given the completely dominant and fully penetrant inheritance pattern of fCJD (Takada and Geschwind, 2013), asymptomatic *PRNP* mutation carriers provide an ideal preclinical model for research, while non-carriers from the same kindred represent the best control group for exploring the disease process. By investigating asymptomatic carriers and patients, previous studies have identified structural and metabolic alterations in individuals with both preclinical and symptomatic CJD.

Diffusion tensor imaging (DTI) is a non-invasive MRI technique that can determine the orientation and integrity of white matter (WM) fibers *in vivo* and has been widely used to show WM changes in the early stages of CJD. Previous DTI studies in CJD cases have demonstrated mean diffusivity (MD) changes in multiple WM tracts (Lee et al., 2012; Wang et al., 2013). A pathological study including 26 patients with CJD applying DTI and post-mortem analysis demonstrated reduced MD and reactive astrocytic gliosis in WM, suggesting WM pathological involvement in symptomatic CJD (Caverzasi et al., 2014b); however, there has been little research investigating WM integrity in the preclinical stage of CJD, although gray matter involvement has been proven (Lee et al., 2009). An FDG-PET study conducted by our research group revealed hypometabolism in the parietal and temporal lobes of the same individual with preclinical CJD (Lu et al., 2020), suggesting that metabolic changes may occur at least 10 years before the estimated onset of clinical symptoms. Therefore, further observations of changes in WM in preclinical CJD are of great significance in studying CJD pathogenesis, as such changes may jointly contribute to disease onset, along with alterations in gray matter.

To investigate changes in WM, we conducted a prospective DTI study in asymptomatic carriers and non-carriers of the *PRNP* G114V mutation from the same fCJD kindred, which were the same individuals as those included in our previous FDG-PET study (Lu et al., 2020). The purpose of the current study was to investigate the patterns of WM changes in the brains of individuals with preclinical CJD.

## Materials and Methods

### Ethics Statement

The study protocols outlined in this manuscript were approved by the Ethics Committee and local Institutional Review Board of Xuanwu Hospital, Capital Medical University, Beijing. All methods and experiments were performed following the relevant guidelines and regulations. All participants enrolled in the study or their guardians signed an informed written consent giving specific approval for this study before the study commenced.

### Participants

Data used in this study were from a Chinese Han fCJD kindred with a G114V mutation in *PRNP*, which has been followed since 2008. Post-mortem analysis was performed in the proband, confirming the diagnosis of CJD (Ye et al., 2008). Details of the clinical findings and genetic analysis of this family have already been published in our FDG-PET study (Liu et al., 2010). Thirteen asymptomatic family members aged > 18 years and one of whose parents was an fCJD patient or G114V mutation carrier were enrolled. Asymptomatic participants were defined as those who did not report any neurological complaints and were normal on neurological examination. Detailed exclusion criteria are provided in our previous FDG-PET study (Lu et al., 2020). Participants were divided into two groups: seven asymptomatic carriers of the *PRNP* V mutation and six non-carriers. All participants underwent clinical examination, neuropsychological assessment, electroencephalogram (EEG) testing, and DTI at baseline. All evaluations were double blind to the genotypes (i.e., neither physicians examining participants nor the participants themselves were aware of their gene mutation status).

Follow-up evaluation was carried out a mean of 2 years after the baseline interview in seven carriers and two non-carriers, and consisted of in-depth clinical examination, neuropsychological assessment, EEG testing, and DTI. Follow-up was not obtained for five non-carriers due to refusal to participate further in the study. All carriers and non-carriers for whom follow-up was obtained had received no treatment for cognitive impairment or neurological symptoms in the previous 2 years.

For comparison with the subset of carriers and non-carriers who were followed-up, from 2018 to 2019, 10 patients with symptomatic CJD who were not from the *PRNP* G114V fCJD kindred were enrolled from the clinic at Xuanwu Hospital, along with age-, and sex-matched healthy controls enrolled from the community. All patients with symptomatic CJD were diagnosed according to the European probable CJD criteria (Zerr et al., 2009). The exclusion criteria were as follows: (1) presence of other causes of cognitive impairment, including small vessel disease, stroke, infection, autoimmune diseases, metabolic diseases, and neurodegenerative diseases other than CJD; (2) incapable of cooperation; (3) history of traumatic brain injury; (4) history of psychosis or congenital mental growth retardation; or (5) contradiction for MRI. Controls were recruited if they had cognitive symptoms, normal general cognitive functioning, and no active neurological or psychiatric disease. The exclusion criteria for healthy controls were the same as those for asymptomatic family members (described above).

### Neurological Assessments

All participants received standardized clinical and cognitive assessments including Mini Mental State Examination (MMSE), Montreal Cognitive Assessment (MoCA), Clinical Dementia Rating (CDR), and neuropsychiatric inventory questionnaire assessments (NPI-Q). Clinicians who performed the assessments were unaware of the mutation status of participants.

### Genetic Analysis for *PRNP* Gene Mutation

Venous blood samples (5 ml) were collected from 10 patients with symptomatic CJD in EDTA anticoagulant vessels and stored at -20°C. A blood extraction kit was used to extract blood DNA. DNA concentration and purity were determined using a NanoDrop2000. Specific primers were used to amplify DNA fragments in the region of the mutation site. Reaction system: 2 × Phanta Max Buffer 12.5 μl, Phanta Max Super-Fidelity DNA Polymerase (1 U/μl) 0.5 μl, dNTP Mix (10 mM each) 0.5 μl, Primer-F (10 μM) 1 μl, Primer-R (10 μM) 1 μl, DNA 1 μl, and water to 25 μl. Reaction procedure: 95°C denaturation 5 min; 95°C denaturation for 30 s, 65°C annealing for 30 s, 72°C extension for 30 s, 25 cycles, each cycle reduced by 0.6; 95°C denaturation for 30 s, 50°C annealing for 30 s, 72°C extension for 1 min, 20 cycles; 72°C extension 10 min. After PCR, 3 μl aliquots of products were analyzed by 2.5% agarose gel electrophoresis, and PCR products were also sequenced using an ABI 3730XL DNA Analyzer.

### Electroencephalogram

All participants received a 2-h EEG at baseline and follow-up using 18-lead electroencephalographic transducers (Micromed, Italy). Electrodes were placed according to the international standard 10–20 system. Conventional single lead, double lead, and sphenoid lead were traced. Eyes closed and deep breathing experiments were performed.

### MRI Acquisition and Imaging Parameters

Magnetic resonance (MR) scanning was performed on a GE Signa PET/MR 3.0 Tesla scanner (GE Healthcare, Milwaukee, WI, United States) in Xuanwu Hospital, Capital Medical University. All 13 asymptomatic family members received their first PET/MR scans in March 2017. Nine of them received follow-up scans in March 2019. All 10 patients with symptomatic CJD received their scans on the same scanner during their hospital stays from 2018 to 2020. DTI data were acquired using a spin echo-echo planar imaging sequence (TR/TE = 16,500/97.6 m) with a *b*-value of 1,000 s/mm^2^, applying diffusion gradients along 30 directions. Seventy axial slices, with no slice gap, were acquired (FOV = 220 × 220 mm^2^, matrix = 112 × 112, slice thickness = 2 mm, and number of excitations = 1).

### Diffusion Tensor Imaging Processing

Diffusion tensor imaging data were preprocessed using the PANDA software package (a pipeline tool for analyzing brain diffusion images, PANDA; http://www.nitrc.org/projects/panda/), which was independently developed by the state key laboratory of cognitive neuroscience and learning of Beijing Normal University. Briefly, preprocessing involved correction of eddy current and head movement, creating a brain mask, and fitting the diffusion tensor model. Outputs were voxel-wise maps of fractional anisotropy (FA) and mean diffusivity (MD). The FA index of DTI is a sensitive neuroimaging measure of degeneration and describes overall WM health, maturation, and organization. MD is another index representing the average dispersion level and dispersion resistance of water molecules overall, which can reflect changes in brain tissue. Higher MD values indicate more free water molecules in the tissue, which influence the information transmission speed to some extent.

### Tract-Based Spatial Statistics Analysis

Tract-based spatial statistics (TBSS) analysis was performed to explore the influence of CJD pathology on WM integrity. In TBSS, all participant FA and MD data were projected onto a mean FA tract skeleton before applying voxel-wise cross-participant statistics. Voxel-wise statistical analyses were conducted using a non-parametric permutation-based inference tool [“randomize,” part of FMRIB Software Library (FSL)] with the general linear model for statistical modeling. Paired *t*-tests, based on voxels, were used for comparisons between carriers at baseline versus follow-up. Student *t*-tests, based on voxels, were used for comparisons between carriers versus non-carriers at baseline, and CJD patients versus healthy controls. The DTI parameters at each voxel were modeled as linear combinations of predictors (five grouping variables) and covariates (age and sex), which were stored in the columns of a “design matrix”; significance thresholds were set at family-wise error (FWE) corrected *P* < 0.05 using the threshold-free cluster enhancement option.

### Statistical Analysis

In this study, SPSS 23.0 software was used to evaluate statistical significance. Differences in age, education, and cognitive scores were assessed using the Student’s *t*-test. Sex differences were assessed using the Chi-square test. Results were considered statistically significant at *p* < 0.05.

Estimated years from expected symptom onset (EYO) values were calculated as the age of the participant at the time of assessment in this study minus the age of the parent at symptom onset; if the parent of the participant had not developed CJD symptoms, the age of the grandparent was used to calculate EYO. For example, if the participant was 35 years old, and the parent’s age at onset was 45 years, then the EYO value would be 10. Parental age at onset was determined using a semi-structured interview in which family members were asked about the age of first progressive cognitive decline.

## Results

### General Characteristics of Study Participants

Asymptomatic family members (*n* = 13) were recruited from the same kindred [the same participants were reported in our previous FDG-PET study (Lu et al., 2020)]. A detailed family tree was published in the report of the FDG-PET and is provided in Supplementary Figure 1 (Lu et al., 2020). In this kindred, nine family members (four males and five females) have passed away due to CJD. Details of the clinical features and examination results of family members are summarized in Supplementary Table 1.

At baseline, seven asymptomatic carriers and six non-carriers from this kindred were examined. Demographic data and neurological assessment scores are summarized in Table 1. There was no significant difference in age, sex, years of education, MMSE score, or MoCA score between the seven asymptomatic carriers and six non-carriers. NPI-Q scores were 0 in all participants. Mean EYO was 13.7 ± 8.4 years in asymptomatic carriers. Details of clinical features and examination results are presented in Supplementary Table 2.

**TABLE 1:** Demographic data of mutation non-carriers and carriers at baseline.

	Non-carriers	Carriers	*p* value
Number of participants	6	7	–
Age (years)	32 ± 8.0	33 ± 10.4	0.77
Sex (male/female)	4/2	2/5	0.29
Years of education	11 ± 3.3	10 ± 2.9	0.80
MMSE	29 ± 1.2	29 ± 0.8	1.00
MoCA	27 ± 2.7	26 ± 5.1	0.77
NPI-Q	0	0	–

*Data are presented as mean ± standard deviation. MMSE, mini-mental state examination; MoCA, Montreal cognitive assessment; and NPI-Q, neuropsychiatric inventory questionnaire.*

All seven carriers and two of the six non-carriers underwent follow-up 2 years after baseline assessment. Neither the carriers nor the non-carriers had developed any neurological symptoms during the 2-year follow-up period. No signs of lesions were detected on EEG or regular MRI. Furthermore, there were no significant differences between baseline and follow-up MMSE and MoCA scores in the seven carriers. NPI-Q and CDR scores were 0 in all participants. Detailed clinical features are shown in Supplementary Table 2. Comparisons of the MRI analysis results of asymptomatic carriers at follow-up and baseline are shown in Supplementary Figure 2.

Among 10 patients with symptomatic CJD, three were familial cases with *PRNP* E200K mutations, and the other seven patients were sporadic cases with no mutations detected. Demographic data and neurological assessment scores for patients with symptomatic CJD are summarized in Table 2. There were no significant differences in age, sex, or years of education between patients with CJD and controls. Details of the clinical features and examination results for the 10 patients with symptomatic CJD are presented in Supplementary Table 3. The results of MRI analysis of patients with CJD and healthy controls are shown in Supplementary Figure 2.

**TABLE 2:** Demographic data of patients with Creutzfeldt–Jacob disease (CJD) and healthy controls.

	CJD patients	Healthy controls	*p* value
Number of participants	10	10	–
Age (years)	61 ± 7.7	56 ± 5.2	0.146
Sex (male/female)	4/6	5/5	0.661
Years of education	10 ± 4.8	11 ± 3.2	0.394
MMSE	4.4 ± 7.5	28 ± 0.8	<0.001
MoCA	1.7 ± 3.9	26 ± 2.7	<0.001
NPI-Q	NA	0	–

*Data are presented as mean ± standard deviation. MMSE, mini-mental state examination; MoCA, Montreal cognitive assessment; NPI-Q, neuropsychiatric inventory questionnaire; and NA, not available.*

### Group Differences in WM Diffusion Metrics Among the Groups

**FIGURE 1:**
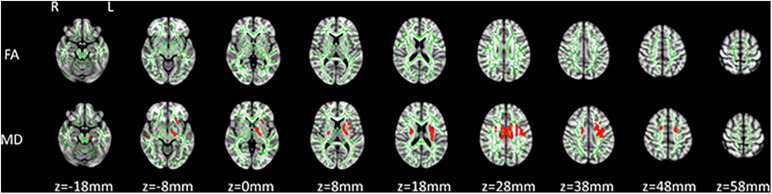
Tract-based spatial statistics (TBSS) analysis of asymptomatic carriers at baseline compared with follow-up. Increased mean diffusivity (MD) was detected by TBSS analysis in asymptomatic prion protein gene (*PRNP)* G114V mutation carriers at follow-up relative to baseline [family-wise error (FWE) correction, *P* < 0.05]. Significant areas of increased MD (red code) in asymptomatic carriers at follow-up relative to baseline are shown with the skeleton (green code).

At baseline, TBSS analysis showed that there was no significant difference in MD and FA between asymptomatic carriers of the *PRNP* G114V mutation and non-carriers (FWE correction, *p* < 0.05). After 2 years, TBSS analysis showed that asymptomatic carriers had increased MD across multiple WM tracts, including the left inferior fronto-occipital fasciculus, left uncinate fasciculus, bilateral anterior thalamic radiation, bilateral cingulate gyrus, bilateral superior longitudinal fasciculus, and bilateral corticospinal tracts (FWE correction, *p* < 0.05; Figure 1). No reduced FA was observed in any WM tract.

**FIGURE 2:**
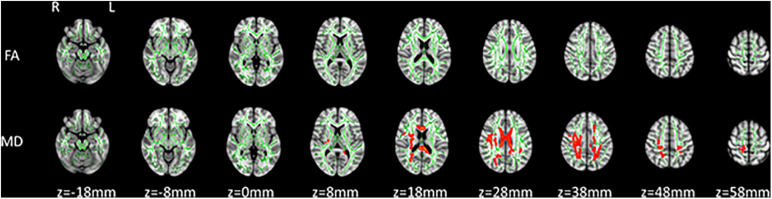
TBSS analysis of symptomatic patients with Creutzfeldt–Jacob disease (CJD) compared with healthy controls. Increased MD was detected in patients with CJD compared with healthy controls (FWE correction, *P* < 0.05). Significant areas of increased MD (red code) in patients with CJD versus healthy controls are shown with the skeleton (green).

Furthermore, significant differences were detected between patients with symptomatic CJD and matched controls (FWE correction, *p* < 0.05; Figure 2). TBSS analysis showed that patients with CJD had increased MD across multiple WM tracts, including the bilateral cingulate gyrus, forceps major, bilateral anterior thalamic radiation, left uncinate fasciculus, and bilateral superior longitudinal fasciculus. No reduced FA was observed in any WM tracts.

**FIGURE 3:**
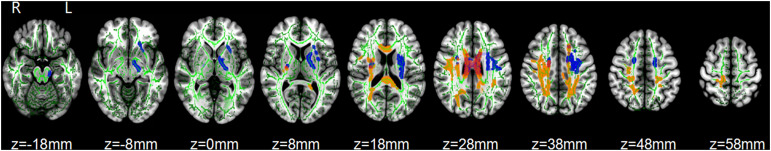
Overlapping patterns of increased MD were found between asymptomatic carriers and patients with CJD. Overlapping areas of increased MD (red code), significant areas of increase MD (blue code) in asymptomatic carriers at follow-up relative to baseline, and significant areas of increased MD (yellow code) in CJD patients versus healthy controls are shown with the skeleton (green code).

**FIGURE 4:**
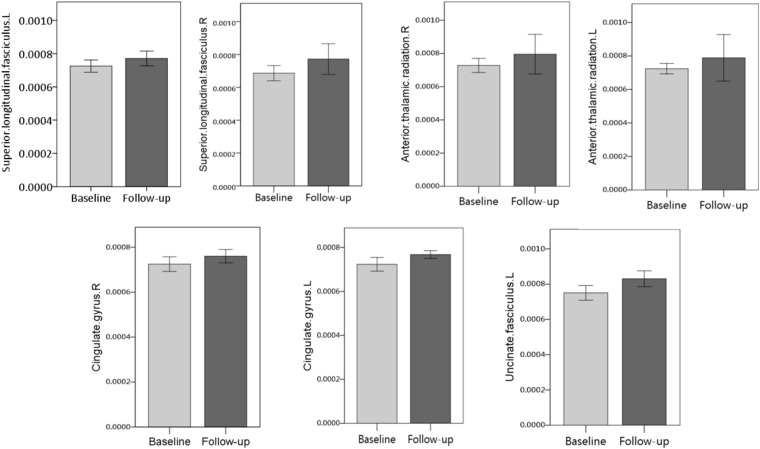
Detailed MD changes (FWE correction, *P* < 0.05) in overlapping white matter (WM) tracts between asymptomatic carriers and patients with CJD are shown. Overlapping patterns were detected in the bilateral superior longitudinal fasciculus, bilateral thalamic radiation, bilateral cingulate gyrus, and left uncinate fasciculus.

Among WM tracts with increased MD in patients with symptomatic CJD, asymptomatic carriers showed overlapping patterns in the bilateral superior longitudinal fasciculus, bilateral anterior thalamic radiation, cingulate gyrus, and left uncinate fasciculus (Figure 3). Details of the observed changes in MD are presented in Figure 4.

## Discussion

In this prospective study, we applied DTI to detect global WM involvement in asymptomatic *PRNP* G114V mutation carriers. We found that changes in WM were characterized by increased MD, accompanied by FA within the normal range. The observed alterations were mainly focused in several WM tracts in asymptomatic carriers, which included patterns overlapping those of patients with symptomatic CJD. Notably, the changes in WM tracts were also correlated with the gray matter metabolic alterations identified in our previous FDG-PET study. To our knowledge, our findings provide the first description of WM abnormalities in the preclinical stage of fCJD, which, together with the gray matter changes revealed by the FDG-PET study, describe pathological imaging changes occurring during the asymptomatic stage of CJD.

In our study, changes were found at follow-up in multiple WM tracts in asymptomatic carriers. Among the detected changes, those in the bilateral superior longitudinal fasciculus, bilateral anterior thalamic radiation, bilateral cingulate gyrus, and left uncinate fasciculus overlapped the patterns detected in patients with CJD. Previous investigations have also detected WM alterations in similar areas, consistent with our data. A DTI study of 26 patients with CJD found MD changes in WM, including in the uncinate fasciculus, superior longitudinal fasciculus, and anterior thalamic radiation (Caverzasi et al., 2014b). Another study applying DTI in 26 patients with CJD identified MD alteration in the cingulate gyrus (Caverzasi et al., 2014a). Notably, related gray matter changes were found in the same participants as those in this study in our previous investigation, which further supports our results.

An FDG-PET study was previously conducted in the same participants included in this investigation. The FDG-PET study focused on gray matter alterations, and the results suggested hypometabolic changes in the parietal and temporal lobes in the preclinical stage of CJD (Lu et al., 2020). Although there is limited evidence supporting WM changes in non-affected carriers, preclinical gray matter changes in the same participants could suggest the involvement of WM. In our FDG-PET study, hypometabolism was observed in the parietal and temporal lobes, while increased MD was found in the superior longitudinal fasciculus, cingulate gyrus, and uncinate gyrus, which are connected to the temporal and parietal lobes (Vogt et al., 2004; Price et al., 2008; Merchant, 2018). These data infer possible metabolic decline in the parietal and temporal lobes, suggesting potential impairment of these WM tracts. Overall, these findings suggest that WM pathological changes occur in patients with preclinical CJD and are potentially correlated with CJD onset.

In our study, increased MD and normal FA were detected in the WM of asymptomatic carriers, while the same alterations were also detected in patients with CJD, and represent pathological imaging changes in the clinical stage of the disease. It may be assumed that the imaging patterns detected in asymptomatic carriers are preclinical pathological changes rather than being secondary to aging; however, the DTI alterations detected differ from those usually reported in patients with CJD (Wang et al., 2013; Caverzasi et al., 2014b), although they are consistent with some other studies with findings that support our results. Notably, a DTI study in patients with familial CJD detected increased MD in several WM tracts, which is consistent with our findings (Lee et al., 2012). Other DTI investigations in patients with sCJD patients also found increased MD in WM (Grau-Rivera et al., 2017). Elevated MD usually appears in patients with demyelination or axonal degeneration, which leads to less restricted movement of water (Lee et al., 2012; Wang et al., 2013; Tromp, 2016); this interpretation is supported by previous histopathological studies, which suggested patchy foci of demyelination in WM (Park et al., 1980; Macchi et al., 1983; Antoine et al., 1996), similar to the pathological changes associated with the second stage of the Braak hypothesis in CJD (Caverzasi et al., 2014b; Iwasaki, 2020). Therefore, we speculate that the increased MD in both asymptomatic carriers and symptomatic patients could be explained by demyelination in the WM. FA reflects the ratio of axial to radial diffusitivity (Wang et al., 2013). We hypothesize that, as the observed WM changes may impair water diffusion in all directions (Matsusue et al., 2004; Wang et al., 2013), which may be attributed to both demyelination and axonal degeneration (Wang et al., 2013), similar alterations in both axial and radial diffusion could account for the observed relative preservation of FA. A previous study of patients with CJD also found that FA was within the normal range, consistent with our findings (Wang et al., 2013). Furthermore, FA preservation was observed in both patients with CJD and asymptomatic carriers, which could further implicate the clinical imaging changes.

A strength of this study is that it shows the earliest known brain changes detected by DTI in the asymptomatic stage of fCJD, which were correlated with the gray matter alterations determined by FDG-PET; however, these results require further confirmation, as the study was limited by the small sample size, since fCJD is a very rare disease. Additionally, the symptomatic patients included in this study were not from the same kindred as the asymptomatic carriers and non-carriers. As our data were collected as part of an ongoing research project, we anticipate that further analyses will be conducted, to investigate the dynamic changes of indices between the asymptomatic and clinical disease stages.

## Conclusion

In conclusion, the preliminary results from our TBSS analysis indicate increased MD in several WM tracts and suggest the involvement of WM integrity in the preclinical stage of fCJD. Overall, this study may provide new insights into CJD pathogenesis.

## Data Availability Statement

The original contributions presented in the study are included in the article/Supplementary Material, further inquiries can be directed to the corresponding author/s.

## Ethics Statement

The studies involving human participants were reviewed and approved by Ethics Committee and local Institutional Review Board of Xuanwu Hospital, Capital Medical University, Beijing. The patients/participants provided their written informed consent to participate in this study.

## Author Contributions

DJ, YCh, ZZ, and LWa were responsible for the study concept and design. CC, HL, LL, JY, ZL, RG, and LWa were responsible for clinical data collection. YCh and ZZ analyzed the image results. DJ, KX, and YCu were the major contributors in writing the manuscript. ZZ and LWu were responsible for critical revision of the manuscript for important intellectual content. All authors contributed to the article and approved the submitted version.

## Conflict of Interest

The authors declare that the research was conducted in the absence of any commercial or financial relationships that could be construed as a potential conflict of interest.

## Abbreviations

WM: white matter
DTI: diffusion tensor imaging
CJD: Creutzfeldt-Jakob disease
fCJD: familial Creutzfeldt-Jakob disease
sCJD: sporadic Creutzfeldt–Jakob disease
PRNP: prion protein gene
EEG: electroencephalogram
TBSS: Tract based spatial statistics
FA: fractional anisotropy
MD: mean diffusivity
MMSE: Mini Mental State Examination
MoCA: Montreal Cognitive Assessment
CDR: Clinical Dementia Rating
NPI-Q: neuropsychiatric inventory questionnaire
FWE: family-wise error
MR: magnetic resonance
FSL: FMRIB Software Library
GLM: general linear model
EYO: Estimated years from expected symptom onset.
