# Supplementary material for: White Matter Integrity Involvement in the Preclinical Stage of Familial Creutzfeldt–Jakob Disease: A Diffusion Tensor Imaging Study
Source: Front Aging Neurosci. 2021 May 19;13:655667. doi: 10.3389/fnagi.2021.655667 (PMC8171061; doi:10.3389/fnagi.2021.655667)
Supplement: Supplementary file 1 [file Table_1.DOCX]

Supplementary Table1. Demographic, clinical features and examinations of deceased fCJD patients from the G114V mutation kindred

| Patient  ID | Sex | Age At Onset | Age At Death | Survival time  (mo) | Clinical features | CSF  14-3-3 | EEG | Brain  MRI | Genotype |
| --- | --- | --- | --- | --- | --- | --- | --- | --- | --- |
| Ⅰ2 | F | 29 | 30 | 12 | NA | NA | NA | NA | NA |
| Ⅱ1 | M | 29 | 30 | 13 | NA | NA | NA | NA | NA |
| Ⅲ2 | F | 63 | 65 | 30 | Cognitive decline, parkinsonism,  myoclonus | Negative | Slowing  PSWCs | Atrophy | G114V |
| Ⅲ3 | M | 45 | 47 | 24 | Cognitive decline, epilepsy,  extrapyramidal symptoms | NA | NA | Atrophy | NA |
| Ⅲ6 | F | 35 | 38 | 30 | Psychiatric symptoms,  cognitive decline, insomnia | NA | NA | NA | NA |
| Ⅲ8 | F | 45 | 48 | 30 | Psychiatric symptoms,  cognitive decline, insomnia,  extrapyramidal symptoms | Negative | Slowing | Atrophy  ribbon | G114V |
| Ⅲ12 | F | 44 | 45 | 15 | Cognitive decline, parkinsonism,  insomnia, psychiatric symptoms | NA | NA | NA | G114V |
| Ⅳ3 | M | 32 | 35 | 36 | Cognitive decline,  insomnia, parkinsonism,  pyramidal symptoms | NA | NA | Negative | G114V |
| Ⅳ15 | M | 27 | 28 | 15 | Cognitive decline,  insomnia, parkinsonism,  psychiatric symptoms | NA | NA | NA | G114V |

M, male; F, female; CSF, cerebrospinal fluid; EEG, electroencephalogram; PSWCs, periodic sharp wave complexes; NA, not available;
