# Supplementary material for: White Matter Integrity Involvement in the Preclinical Stage of Familial Creutzfeldt–Jakob Disease: A Diffusion Tensor Imaging Study
Source: Front Aging Neurosci. 2021 May 19;13:655667. doi: 10.3389/fnagi.2021.655667 (PMC8171061; doi:10.3389/fnagi.2021.655667)
Supplement: Supplementary file 2 [file Table_2.DOCX]

Supplementary Table 2. Demographic and neurological assessments of the family participants from baseline to follow-up

| ID | Sex | PRNP | Age At Baseline  (year) | EYO* At Baseline  (year) | MMSE | | MoCA | | CDR | | NPI-Q | |
| --- | --- | --- | --- | --- | --- | --- | --- | --- | --- | --- | --- | --- |
|  |  |  |  |  | Baseline | Follow-up | Baseline | Follow-up | Baseline | Follow-up | Baseline | Follow-up |
| Ⅳ-2 | F | G114V | 45 | -18 | 28 | 23 | 15 | 15 | 0 | 0 | 0 | 0 |
| Ⅳ-8 | F | G114V | 46 | 1 | 30 | 30 | 27 | 29 | 0 | 0 | 0 | 0 |
| Ⅳ-10 | F | G114V | 42 | -3 | 29 | 30 | 29 | 27 | 0 | 0 | 0 | 0 |
| Ⅳ-19 | F | G114V | 28 | -17 | 30 | 30 | 30 | 30 | 0 | 0 | 0 | 0 |
| Ⅴ-6 | M | G114V | 28 | -17 | 29 | 29 | 27 | 28 | 0 | 0 | 0 | 0 |
| Ⅴ-8 | M | G114V | 25 | -18 | 28 | 29 | 27 | 29 | 0 | 0 | 0 | 0 |
| Ⅴ-10 | F | G114V | 21 | -24 | 29 | 30 | 29 | 30 | 0 | 0 | 0 | 0 |
| Ⅳ-5 | M | 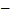 | 40 | NA | 27 | 29 | 23 | 27 | 0 | 0 | 0 | 0 |
| Ⅳ-11 | M | 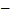 | 40 | NA | 30 | NA | 29 | NA | 0 | 0 | 0 | 0 |
| Ⅳ-14 | F | 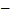 | 36 | NA | 30 | NA | 24 | NA | 0 | 0 | 0 | 0 |
| Ⅳ-17 | M | 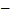 | 31 | NA | 30 | 29 | 29 | 29 | 0 | 0 | 0 | 0 |
| Ⅴ-1 | F | 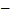 | 24 | NA | 28 | NA | 28 | NA | 0 | 0 | 0 | 0 |
| Ⅴ-2 | M | 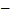 | 23 | NA | 29 | NA | 29 | NA | 0 | 0 | 0 | 0 |

PRNP, prion-related protein gene; EYO, estimated years from expected symptom onset; MMSE, mini-mental state examination;

MoCA, Montreal Cognitive Assessment; CDR, Clinical Dementia Rating; NPI-Q, Neuropsychiatric Inventory Questionnaire;

-, negative; NA, not available

*：“-”, The age of the participant at the time of the study has not been exceeded the estimated age from expected symptom onset.
