# Supplementary material for: White Matter Integrity Involvement in the Preclinical Stage of Familial Creutzfeldt–Jakob Disease: A Diffusion Tensor Imaging Study
Source: Front Aging Neurosci. 2021 May 19;13:655667. doi: 10.3389/fnagi.2021.655667 (PMC8171061; doi:10.3389/fnagi.2021.655667)
Supplement: Supplementary file 3 [file Table_3.DOCX]

Supplementary Table3 Demographic, clinical features and examinations of CJD patients

| Patient  ID | Sex | Age At Onset | Survival time  (mo) | Family  history | Clinical features | CSF  14-3-3 | EEG | DWI  Hyperintensities | Genotype |
| --- | --- | --- | --- | --- | --- | --- | --- | --- | --- |
| 1 | M | 69 | 22 | - | Cognitive decline, parkinsonism,  akinetic mutism | NA | PSWs | Cortical,caudate | - |
| 2 | M | 70 | 3.5 | - | Cognitive decline, myoclonus,  psychiatric symptoms | NA | NA | Cortical | - |
| 3 | F | 57 | 5 | - | Cognitive decline,  pyramidal symptoms,  extrapyramidal symptoms,  psychiatric symptoms,  ataxia, myoclonus | - | Slowing | Cortical,caudate | - |
| 4 | F | 61 | 12.5 | - | Cognitive decline,  extrapyramidal symptoms,  visual deficits, akinetic mutism | + | NA | Cortical,caudate  putamen | - |
| 5 | M | 52 | 4.5 | - | Cognitive decline, parkinsonism,  myoclonus, akinetic mutism | - | PSWs | Cortical,caudate | - |
| 6 | F | 60 | 2 | - | Cognitive decline, myoclonus,  extrapyramidal symptoms ,  pyramidal symptoms,  psychiatric symptoms,  akinetic mutism | - | Slowing | Cortical,caudate  putamen | - |
| 7 | F | 74 | 18.5 | - | Cognitive decline | - | Slowing | Cortical | - |
| 8 | M | 57 | 11 | - | Cognitive decline, insomnia,  extrapyramidal symptoms, myoclonus, akinetic mutism | NA | PSWs | Cortical | E200K |
| 9 | F | 53 | 10 | + | Cognitive decline,  extrapyramidal symptoms,  myoclonus, ataxia | + | PSWs | Cortical | E200K |
| 10 | M | 56 | 12 | + | Cognitive decline, ataxia,  insomnia, myoclonus,  extrapyramidal symptoms ,  pyramidal symptoms,  psychiatric symptoms | - | PSWs | Caudate, putamen | E200K |

M,male; F, female; CSF, cerebrospinal fluid; EEG, electroencephalogram; DWI, diffusion weight imaging; PSWCs, periodic sharp wave complexes; NA, not available; -, negative; + ,positive
