# Supplementary figures and images for: White Matter Integrity Involvement in the Preclinical Stage of Familial Creutzfeldt–Jakob Disease: A Diffusion Tensor Imaging Study
Source: Front Aging Neurosci. 2021 May 19;13:655667. doi: 10.3389/fnagi.2021.655667 (PMC8171061; doi:10.3389/fnagi.2021.655667)

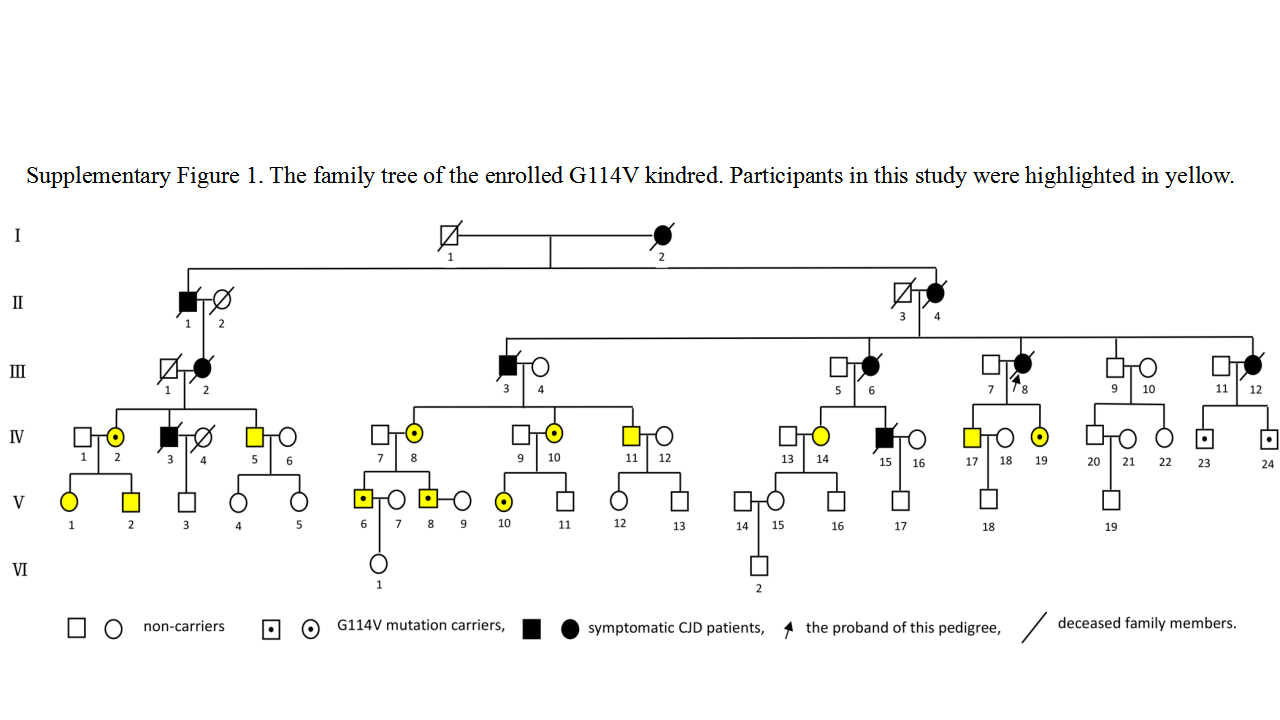

Supplement: Supplementary file 4 [file Image_1.tif]

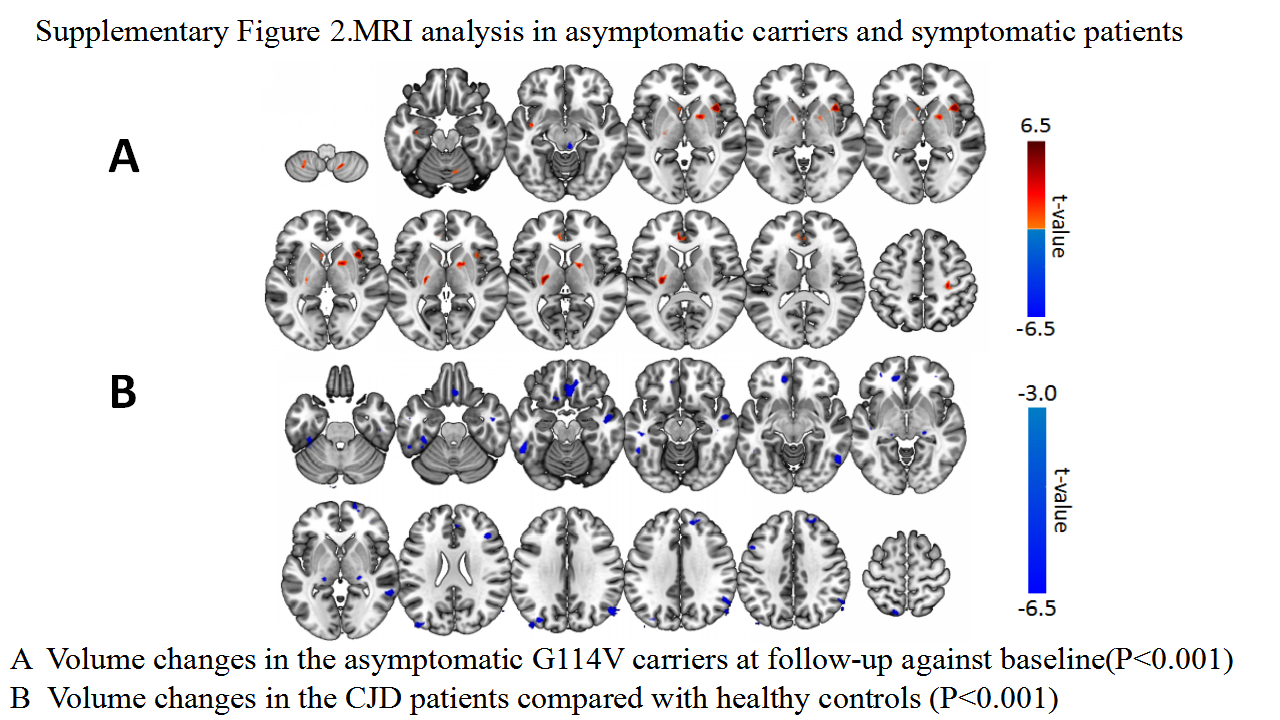

Supplement: Supplementary file 5 [file Image_2.tif]
